# Multi‐omics analysis of ion channels expressed by chicken embryo vestibular type I and type II hair cells

**DOI:** 10.1113/EP094078

**Published:** 2026-09-15

**Authors:** Giulia Cheli, Roberta Giunta, Francesca Stella, Eleonora Guidi, Umberto Laforenza, Mariarosa Polimeni, Giancarlo Russo, Francesca Borgo, Lazarevic Dejan, Sergio Masetto

**Affiliations:** ^1^ Department of Brain and Behavioral Sciences University of Pavia Pavia Italy; ^2^ Center for Omics Sciences IRCCS San Raffaele Institute Milan Italy; ^3^ Vita‐Salute San Raffaele University Milan Italy; ^4^ Department of Molecular Medicine University of Pavia Pavia Italy; ^5^ Department of Public Health, Experimental and Forensic Medicine University of Pavia Pavia Italy

**Keywords:** hair cell, ion channel, patch clamp, patch‐sequencing, transcriptomics, vestibular hair cell

## Abstract

Balance and gaze rely on the rapid and accurate detection and signalling of head movements by vestibular type I and type II hair cells. Signal transduction and transmission involve several types of ion channels, which are acquired progressively during hair cell differentiation and whose identity is known only in part. In the present study, we adopted a multi‐omics approach to identify the molecular nature of ion channels expressed by chicken vestibular hair cells between embryonic day 15 and embryonic day 21, the day of hatching, when both type I and II hair cells appear functionally mature. Type I hair cells are characterized by the expression of a low‐voltage‐activated outward rectifying K^+^ current, termed *I*_KL_. In adult mice, *I*_KL_ is carried by the K_V_1.8 α‐subunit, although it is still debatable whether the K_V_7.4 α‐subunit contributes to *I*_KL_ in immature mouse type I hair cells. In this study, no effect of linopirdine, a specific blocker of K_V_7 channels, was found in either immature or mature type I or II hair cells. Furthermore, whole‐organ RT‐PCR and transcriptome analysis showed no expression of the K_V_7.4 α‐subunit transcript, while detecting the transcript for the K_V_1.8 subunit. Its expression in type I and II hair cells was finally confirmed by patch‐sequencing experiments. Further analysis of the transcriptomes of individual hair cells revealed the presence of transcripts for several other α and auxiliary subunits of Na^+^, K^+^ and Ca^2+^ channels, which are, presumably, responsible for the ionic currents recorded in both birds and mammals.

## Abstract (highlights)

- **What is the central question of this study?**
  What are the molecular identities of the ion channels expressed by vestibular type I and type II hair cells?
- **What is the main finding and its importance?**
  By using the patch‐sequencing technique, we have provided the first single‐cell transcriptomic datasets from electrophysiologically identified vestibular type I and type II hair cells. Defining the nature of ion channels expressed by vestibular hair cells is essential to understanding the mechanism of signal processing, assessing the cause of peripheral vestibular disorders and developing effective pharmacological treatments.

## 1 INTRODUCTION

In the vestibular organs of amniotes (reptiles, birds and mammals), head movements are sensed and signalled to the nervous system by two types of sensory cells, named type I and type II hair cells, which differ in morphology, innervation and electrophysiological properties (Eatock & Songer, 2011). Type I and II hair cells begin to differentiate early during prenatal development, through the timely and orchestrated addition, regulation and replacement of distinct voltage‐gated ion channels (Corns et al., 2014). In chicken embryos (Masetto et al., 2000), an inward Ca^2+^ current (*I*_Ca_) and a slow outward rectifying K^+^ current (*I*_KV_) are already present at embryonic day (E)10, followed by a rapid transient K^+^ current of A‐type (*I*_KA_) at E12, a Ca^2+^‐dependent K^+^ current (*I*_KCa_) at E14, a hyperpolarization‐activated cationic current (*I*_h_) around E16, and the fast inward rectifying K^+^ current (*I*_Kir_) around E19. From E17, some hair cells start to express *I*_KL_, the signature current of mature type I hair cells (Rennie & Correia, 1994; Rüsch & Eatock, 1996). A voltage‐dependent Na^+^ current (*I*_Na_) was also found in most hair cells from E14 (Masetto et al., 2003). Close to hatching (E21), hair cell complements of ion currents appear similar to those reported for the mature avian crista (Masetto & Correia, 1997).

In comparison, the prenatal development of voltage‐gated ion channels in hair cells has been less studied in mammals. Except for a study on mouse embryos showing the prenatal appearance of *I*_KV_ at E13, *I*_KA_ at E14, *I*_Na_ and *I*_Kir_ at E15, and *I*_KL_ at E18 (Géléoc et al., 2004), most studies have been performed on neonatal rodents and show that final maturation of many type I and II hair cells occurs postnatally (Eatock & Hurley, 2003; Li et al., 2009; Rüsch et al., 1998). Indeed, *I*_h_ was not found in prenatal mouse hair cells (Géléoc et al., 2004), first appearing at postnatal day (P)3 (Rüsch et al., 1998).

The above complex developmental scenarios, combined with the limited availability of specific drugs, has made it difficult to identify the molecular substrate of the ionic currents recorded from developing and mature type I and II vestibular hair cells.

In the mouse genome, 40 genes are known to encode pore‐forming (α) subunits of voltage‐gated K^+^ channels (identical to those in humans; Jegla et al., 2009), whereas most extant birds possess 38 such genes (which, compared with mice, lack the genes for the K_V_1.7, K_V_3.3 and K_V_4.1 α‐subunits but include the gene for K_V_7.1; Jiang et al., 2025), classified into 12 subfamilies. These subunits can assemble into homo‐ or heterotetramers and often associate with various auxiliary subunits to form functional channels that differ in their functional properties and pharmacology.

In the present study, we used a multi‐omics approach to investigate the nature of voltage‐gated ion channels in type I and II hair cells of the vestibular crista of the chicken embryo in the last week of development. Our results show that the K_V_7.4 α‐subunit, reported to sustain *I*_KL_ in immature but not mature mouse type I hair cells (Holt et al., 2007), was not expressed in type I and type II hair cells, based on the lack of effect of the specific blocker, linopirdine. Type II hair cells were included in the analysis because it has been reported that selective pharmacological blockers of K_V_7.1 to K_V_7.4 subunits reduce the delayed‐rectifier K^+^ current in developing type II hair cells of the rat utricle (Hurley et al., 2006) and the residual K^+^ current in mature type I and type II hair cells of *Kcna10* ^−/−^ mice, which lack the K_V_1.8 (Martin et al., 2024). Consistent with patch‐clamp recordings, RT‐PCR (E16 and E20) and transcriptomic (E16) analysis of the whole vestibular organ (semicircular canal crista) showed the absence of the mRNA for the K_V_7.4 subunit. The latter approach, however, revealed the expression at these time points of the mRNA for the K_V_1.8 α‐subunit, which is responsible for *I*_KL_ in adult mouse type I hair cells (Martin et al., 2024). Because it cannot be ruled out that non‐sensory cells express the K_V_1.8 α‐subunit and because there are no specific blockers for the K_V_1.8 α‐subunit that can be tested in patch‐clamp recordings, we envisioned an additional approach, as follows. The advent of next‐generation sequencing has made it possible to obtain the complete transcriptome from a single cell, overcoming the limitations of bulk sequencing. We therefore performed, for the first time, patch‐sequencing (Patch‐seq) experiments on vestibular hair cells. Patch‐seq is a recent, technically challenging but powerful technique that combines whole‐cell patch‐clamp recording with RNA sequencing in the same cell (Cadwell et al., 2016; Lipovsek et al., 2020). We obtained high‐quality results from an E20 type I and an E19 type II hair cell, showing the expression of the K_V_1.8 α‐subunit, while confirming the absence of the K_V_7.4 α‐subunit, in both. Further analysis of their transcriptome revealed the presence of mRNA for the Na_V_1.7 α‐subunit, accounting for *I*_Na_, the Ca_V_1.3 α‐subunit alone for *I*_Ca_, previously unreported K^+^ channel α‐subunits (K_V_1.6, K_V_4.2 and K_Ca_1.1), and other proteins characteristic of hair cells.

## 2 MATERIALS AND METHODS

### 2.1 Ethical approval

Animal procedures on chicken embryos conform to the guidelines from Directive 2010/63/EU of the European Parliament on the protection of animals used for scientific purposes. The study protocol did not require the authorization of the Ministry of Health, as indicated in the Legislative Decree 4 March 2014, no. 26, following the implementation of Directive (2010)/63/EU on the protection of animals used for scientific purposes.

### 2.2 Surgical dissection

Fertilized chicken (Isa Brown) eggs of both sexes were obtained by a local supplier and incubated at 37.7°C. All experiments were performed in situ on hair cells in whole vestibular epithelia dissected from chicken embryos between E16 and E21.

Once removed from the eggs, embryos were decapitated following brief anaesthesia with 2‐bromo‐2‐chloro‐1,1,1‐trifluoroethane (halothane), and semicircular canals with their ampullae containing the sensory crista were dissected in chilled extracellular solution (mM): 145 NaCl, 3 KCl, 2 CaCl_2_, 0.6 MgCl_2_, 5.6 d‐glucose, 15 HEPES; pH adjusted to 7.4 with NaOH; osmolality ∼310 mosmol kg^−1^) under a stereomicroscope (Leica MZ95).

### 2.3 Whole‐cell electrophysiology

Following exposure of the sensory crista by cutting the edge of the ampulla, the dissected organ was immobilized at the bottom of the recording chamber by a nylon mesh glued to a silver ring. Hair cells were viewed using an upright microscope (Olympus BX51WI, Evident Corporation) equipped with a ×60 water immersion objective. The precise position of the recorded hair cell within the sensory epithelium was not determined. Hair cells in the sensory organs were approached from their basolateral membrane. Cellular debris was gently removed using the tip of a patch pipette to facilitate access (for details, see Contini et al., 2012), and recordings were obtained with a second patch pipette. A major complication when recording from type I hair cells is the presence of the calyx, which almost completely encloses the basolateral membrane of the type I hair cells. In adult mice, even after piercing by the tip of the patch pipette the calyx remains firmly attached to the hair cells, which can be inferred by the accumulation of K^+^ in the calyceal synaptic cleft during a large outward K^+^ current elicited in the hair cell (Spaiardi et al., 2017, 2025). In the chicken embryo, intercellular K^+^ accumulation was also observed, confirming the parallel development of *I*_KL_ and the calyx previously reported (Masetto et al., 2000).

Whole‐cell patch‐clamp experiments were conducted at room temperature (22°C) using an Axopatch 200B amplifier (Molecular Devices, San Jose, CA, USA). Voltage protocol application and data acquisition were controlled by pClamp v.11.2 software using a Digidata 1550B board (Molecular Devices, San Jose, CA, USA). Voltage‐clamp recordings were low‐pass filtered at 5 kHz (four‐pole Bessel) and sampled at 50 kHz. Data analysis was performed using Clampfit (Molecular Devices, San Jose, CA, USA) and OriginPro 9.0 software (OriginLab, Northampton, MA, USA).

Patch pipettes (4–7 MΩ) were pulled from borosilicate glass capillaries (World Precision Instruments, Hitchin, UK). The patch pipette filling solution contained (mM): 134 KCl, 2 MgCl_2_, 1 CaCl_2_, 11 EGTA and 10 HEPES. pH was adjusted to 7.4 using KOH (osmolality: 290 mosmol kg^−1^).

The voltages presented in the figures and text were not adjusted for the liquid junction potential with the intracellular solution (resulting in a 3 mV negative potential inside the pipette); hence, nominal voltages are reported. Leak current subtraction was not performed.

Linopirdine (Merck, Darmstadt, Germany) was dissolved initially in DMSO, then introduced into the extracellular solution to achieve a final concentration of 10 or 100 µM L^−1^. In preliminary experiments, we assessed that the quantity of DMSO added to the extracellular solution (1%) did not by itself affect the electrophysiological responses recorded from the hair cells.

Linopirdine was perfused by a multi‐barrelled pipette placed ∼50 µm from the target cell. Each cell was initially recorded in control conditions, then exposed to linopirdine. The drug was washed off by 60 s of superfusion with bath solution and exchange of the bath solution using a peristaltic pump. The preparation was changed after each perfusion to avoid recording from cells that had already been exposed to the drug.

Current responses were analysed offline. Current–voltage (*I*–*V*) relationships were built by normalizing peak and steady‐state current amplitudes to the maximal current measured in control conditions and plotting normalized values against membrane potential.

### 2.4 RNA extraction and RT‐PCR

For gene expression analysis, cells were isolated from 64 semicircular canal cristae from chicken embryos at E16 and E20, and pooled. Total RNA was extracted using the ReliaPrep™ RNA Miniprep Systems (Z6010, Promega, Italy), following the manufacturer's instructions. Complementary DNA (cDNA) was synthesized using the M‐MLV Reverse Transcriptase M1701 (Promega, Milan, Italy) with random hexamers in a total reaction volume of 20 µL, as previously described (Pellavio et al., 2022). Primers for the target and reference genes (Table 1) were validated for specificity using BLAST analysis. Quantitative PCR (qPCR) was performed on the Applied Biosystems StepOnePlus™ Real‐Time PCR System (Thermo Fisher Scientific, Monza, Italy) using Hot Disco Taq 2X Master Mix (catalogue no. 257685; Biosigma S.p.A., Dominique Dutscher Group, Cona, Venice, Italy). The cycling conditions were as follows: initial denaturation at 95°C for 15 min, followed by 40 cycles of denaturation at 95°C for 30 s, annealing at 58°C for 45 s and extension at 72°C for 60 s. No‐template controls were included to monitor contamination. Melt‐curve analysis confirmed the presence of a single amplicon and the absence of primer dimers. PCR products were also resolved on an agarose gel, stained with ethidium bromide, and visualized using the iBright™ CL1000 Imaging System with the accompanying software (Thermo Fisher Scientific, Monza, Italy). Molecular weights were determined by comparison with the DNA Molecular Weight Marker VIII (Roche Molecular Biochemicals, Italy). Samples were expressed as ΔCt values, obtained as follows:

$$\Delta Ct=Ct_{\mathrm{target}}-Ct_{\mathrm{geo}},$$

where $Ct_{\mathrm{geo}}$ is the geometric mean of the Ct values of the two housekeeping genes, Actin Beta (*Actb*) and Hypoxanthine Phosphoribosyltransferase 1 (*Hprt1*), calculated using the formula below:

$$\;Ct_{\mathrm{geo}}=\sqrt{Ct_{Actb}\;\times \;Ct_{Hprt1}}.$$

**TABLE 1:** Primer sequences used for RT‐PCR.

Gene	Gene	Primer sequence		Size (bp)
Actin beta	*ACTB*	F	5′‐CCACACCCCTGTGATGAAACA‐3′	280
		R	5′‐CCTTCACAGAGGCGAGTAACT‐3′	
Calbindin 1	*CABI*	F	5′‐TGGCACTGAAATCCCACTGAA‐3′	118
		R	5′‐TAACATGCCAAGACCAAGGCT‐3′	
Calretinin	*CARE*	F	5′‐TTTCAGGGGATGAAGCTGTCC‐3′	121
		R	5′‐TCTCGTAGAGGTCCTTCAGCA‐3′	
Myosin VIIA	*MYOS*	F	5′‐TGCTTGTCTCATCTTCCACCC G‐3′	111
		R	5′‐CCCTCTACTCTCCCAGACACA‐3′	
Hypoxanthine phosphoribosyltransferase 1	*HPRT1*	F	5′‐TGCACTGATCTGAAGTGCAGG‐3′	147
		R	5′‐GCTGCAAAGATGCTTGTCTGA‐3′	
Potassium voltage‐gated channel subfamily Q member 2	*KCNQ2*	F	5′‐GCTAGGGGAATGCTCTGAACC‐3′	125
		R	5′‐ATTTCTGTGCTCCCTGGGTTG‐3′	
Potassium voltage‐gated channel subfamily Q member 4	*KCNQ4*	F	5′‐GGCCAAGAGGAAGTTCAAGGA‐3′	337
		R	5′‐CAGACTGAAGGAGTTGGCTGA‐3′	
Potassium voltage‐gated channel subfamily Q member 5	*KCNQ5*	F	5′‐GCTGCCAGTTGCAAGATACAC‐3′	144
		R	5′‐ACAATGTGACAGTAGTGGCGT‐3′	
Potassium voltage‐gated channel subfamily B member 1	*KCNB1*	F	5′‐CTGACTCTGGGAGGTTTTCCC‐3′	135
		R	5′‐TGCCTTCACAGCTTGGGTTTA‐3′	
Potassium voltage‐gated channel subfamily A member 3	*KCNA3*	F	5′‐ATGTTTCCACCTTTTGCACCG‐3′	113
		R	5′‐GCATCACTCCGGTCCGAATTA‐3′	
Potassium voltage‐gated channel subfamily A member 4	*KCNA4*	F	5′‐TTGCTTTACCAGTGCCAGTGA‐3′	121
		R	5′‐TTTGTTGGGAGGTAAGGGCAA‐3′	
Potassium voltage‐gated channel subfamily A member 10	*KCNA10*	F	5′‐TTTGAGTACCCCGAAAGCTCC‐3′	115
		R	5′‐CCTCTCTGAACTCTGGCAAGG‐3′	
Potassium voltage‐gated channel modifier subfamily G member 4	*KCNG4*	F	5′‐TTCTTTGCAACACCTTGAGCC‐3′	100
		R	5′‐CTTCCTCTGTATGCCTGGGTG‐3′	
Potassium voltage‐gated channel subfamily H member 7	*KCNH7*	F	5′‐ACAGCCTCAGAAGACAACCTG‐3′	112
		R	5′‐AGGAAGGGTGCCTGATTGATG‐3′	
Potassium inwardly rectifying channel subfamily J member 2	*KCNJ2*	F	5′‐TCCCGGAAAAGACAAAGGGTC‐3′	129
		R	5′‐TTACAATGGTCGTGAGCAGCC‐3′	
Potassium inwardly rectifying channel subfamily J member 5	*KCNJ5*	F	5′‐GCTCCTACATGGACACAGAGG‐3′	128
		R	5′‐GCACACAGGAGTGTTGGTTTC‐3′	
Potassium inwardly rectifying channel subfamily J member 11	*KCNJ11*	F	5′‐AGAGCATTATGGACACCGTGC‐3′	131
		R	5′‐GCCCTAACACAGTTTCCCAGT‐3′	
Hyperpolarization activated cyclic nucleotide gated potassium channel 1	*HCN1*	F	5′‐CTTGCCAAGGGATTCTTCTGC‐3′	116
		R	5′‐CTCAGTTTGTCGCAAGTCTGC‐3′	

*Note*: Annealing temperature used was 58°C.

Abbreviations: F, forward; R, reverse.

Relative expression was calculated with the ΔΔCt method, which was then applied to determine the fold change in gene expression according to the following formula:

$$\mathrm{Fold}\;\mathrm{change}=-2^{\left(\Delta Ct_{E20}-\Delta Ct_{E16}\right)}.$$

### 2.5 Whole‐organ transcriptomics

Ampullary cristae were dissected from 22 chicken embryos at E16, and total RNA was extracted using QIAzol Lysis Reagent (Qiagen, Italy). Bulk RNA sequencing was carried out on a single biological sample processed at 500 pg RNA input level. Reverse transcription and amplification were performed with the SMART‐Seq Low Input RNA Kit (Takara Bio). Amplified cDNA was subsequently fragmented, ligated to adapters, and indexed using the Nextera XT DNA Library Preparation Kit (Illumina). Final libraries were sequenced on an Illumina NovaSeq 6000 platform, producing paired‐end reads in FASTQ format according to standard Illumina protocols. Sequencing reads were processed with the Illumina DRAGEN Bio‐IT Platform. A custom *Gallus gallus* reference genome (Ensembl GRCg7b assembly) was generated with the DRAGEN Reference Builder, incorporating both FASTA and GTF annotation files. Alignment was performed using the DRAGEN RNA pipeline under default settings, and all downstream analyses followed the standard DRAGEN workflow. The full transcriptomic dataset is available at NCBI Gene Expression Omnibus (GEO) data repository (GSE336620).

### 2.6 Patch‐sequencing

#### 2.6.1 Patch‐sequencing preparation procedures

To ensure RNase‐free conditions for Patch‐seq experiments (Lipovsek et al., 2020), all materials, including glassware, spatulas, stir bars and pipettes, were autoclaved and subsequently sterilized by exposure to ultraviolet irradiation. Work surfaces were decontaminated with 70% ethanol (Sigma‐Aldrich, St. Louis, MO, USA) prepared in DEPC‐treated water (Invitrogen, Waltham, MA, USA) and treated with RNaseZap (Sigma‐Aldrich, St. Louis, MO, USA) to prevent RNase contamination.

The extracellular solution maintained the same composition as described above, whereas the intracellular solution was prepared with a lower KCl concentration (106 mM). This adjustment was necessary because the addition of RNase inhibitor (Qiagen, Venlo, The Netherlands) and glycogen (Thermo‐Scientific, Waltham, MA, USA) increased the final osmolarity of the solution by ∼50 mosmol L^−1^. However, presumably because the osmotic difference between the extracellular and intracellular solutions was not perfectly balanced, we noticed that the current recordings were less stable, which is why in Figure 8 the baseline currents at the holding voltage of −70 mV have been adjusted to zero.

Both solutions were filtered through sterile 0.2 µm filters, then aliquoted and stored at −4°C until use.

On the day of the experiment, 2 µL of RNase inhibitor and 0.25 µL of glycogen were added to 250 µL of intracellular solution to preserve RNA integrity.

Patch pipettes (≤5 MΩ), were pulled from borosilicate glass capillaries (World Precision Instruments, Hitchin, UK) and filled with 2 µL of intracellular solution using gel‐loading tips to ensure accurate loading of the small volume.

#### 2.6.2 Patch‐clamp recording and cytoplasm extraction

To minimize RNA contamination, these experiments were performed in a different room, with a dedicated set‐up equipped with an upright microscope (Zeiss 2 FS plus, Göttingen, Germany) with a ×67 water immersion objective, an Axopatch 200B amplifier (Molecular Devices, San Jose, CA, USA) and a Digidata 1440A board (Molecular Devices, San Jose, CA, USA). Voltage protocols and data acquisition were controlled by pClamp v.10.3 software. Current recordings were low‐pass filtered at 5 kHz (four‐pole Bessel) and sampled at 50 kHz. Whole‐cell patch‐clamp experiments were conducted at room temperature (22°C) on hair cells in situ in vestibular sensory epithelia dissected from chicken embryos between E19 and E20 (see Section 2.2).

The Patch‐seq technique requires the establishment of a stable giga‐ohm seal not only to ensure high‐quality electrophysiological recordings, but also to preserve the integrity of cytoplasmic material and to avoid contamination from the extracellular environment. Once whole‐cell configuration was achieved, a recording protocol was initiated to identify the hair cell type based on electrophysiological properties.

At the end of the recording, negative pressure was reapplied until the cell showed a marked reduction in size; this phenomenon indicated that the cytoplasm had been aspirated into the patch pipette. Once this had been achieved, the pipette tip was moved into contact with the nucleus to perform ‘corking’, a step aimed at reducing contamination from the extracellular solution and improving RNA yield and transcriptomic data quality (Lipovsek et al., 2020). During this phase, negative pressure was maintained and the access resistance monitored; variation of access resistance was considered indicative of seal formation onto the nucleus.

After confirming the presence of the nucleus at the pipette tip, the pipette was slowly and gradually retracted to detach the hair cell from the surrounding tissue. Before withdrawing the pipette from the bath solution, the absence of debris attached to the cell outer surface was checked carefully by focusing with the objective. The aspirated cellular content was then expelled into a collection tube containing 2 µL of lysis buffer, placed on a metal plate kept in contact with dry ice. Samples were subsequently stored at −80°C until sequencing. The cytoplasm extraction procedure lasted no longer than 5 min to reduce the risk of RNA degradation.

Each cell was assigned a qualitative (not numerical) score, and only samples meeting the predefined criteria were processed for sequencing (for better details, see Lipovsek et al., 2020: figure 3). Criteria included: maintenance of the electrical seal throughout the entire extraction procedure, absence of debris on the external pipette surface, no contact of the pipette tip with external surfaces before immersion in lysis buffer, and total extraction time <10 min.

#### 2.6.3 Sample preparation and library construction

Based on the scores obtained during cytoplasmic extraction, a total of 12 hair cells were selected for transcriptome analysis. Two of them, however, showed human DNA contamination, and eight showed suboptimal transcript counts. Ultimately, only one type I and one type II hair cell were obtained with sufficient RNA quality to generate high‐confidence sequencing data. However, low‐expressing transcripts might be underrepresented owing to inefficient RNA capture and suboptimal reverse transcription efficiency, compounded by variability in RNA integrity. Consequently, absence of detection cannot be interpreted with certainty as true biological absence, meaning that false negatives remain a significant concern. We attempted to overcome this limitation by comparing single‐cell data with bulk data for those transcripts expected to be present in hair cells based on the literature.

Library preparation was conducted directly on cell lysates immediately following recovery using a lysis buffer. Full‐length cDNA was generated using the SMART‐Seq v.4 Ultra Low Input RNA Kit (Takara Bio USA, Ann Arbor, MI, USA) according to the manufacturer's instructions, with 18 cycles of PCR amplification. This technology leverages the template‐switching activity of reverse transcriptase to enrich for full‐length cDNAs and attach defined adapters to both ends of the first‐strand cDNA, ensuring accurate representation of the original mRNA transcripts, including the 5′ ends.

The quality and size distribution of the resulting cDNA were assessed using the High Sensitivity D5000 ScreenTape Assay on a 4200 TapeStation System (Agilent Technologies). Sequencing libraries were subsequently prepared using the Illumina Prep kit (Illumina, San Diego, CA, USA) following the manufacturer's protocol, with the tagmentation step reduced to 2 min. This procedure uses a transposase‐based tagmentation mechanism simultaneously to fragment the cDNA and ligate sequencing adapters. Final libraries were quantified via TapeStation, pooled at a 1 nM concentration, and sequenced (1 × 100 bp) on the Illumina NovaSeq 6000 platform. The full transcriptomic dataset is available at NCBI Gene Expression Omnibus (GEO) data repository (GSE336620).

### 2.7 Statistics

For electrophysiological recordings, statistical analysis was performed by Prism GraphPad v.8.0 Software (San Diego, CA, USA). Following Shapiro–Wilk normality test, a mixed‐effects model was applied to analyse repeated‐measures data with missing values, specifically for the analysis of outward currents in type I and type II hair cells. Tukey's test was used for *post hoc* comparisons. To assess the effect of linopirdine on inward currents in type II hair cells, data were first tested for normality using the Shapiro–Wilk test, then analysed using either Student's paired *t‐*test or a Wilcoxon matched‐pairs signed rank test for parametric and non‐parametric data, respectively.

For RT‐PCR experiments, all statistical analyses were performed on ΔCt values, as recommended by the MIQE (Minimum Information for Publication of Quantitative Real‐Time) guidelines (Bustin et al., 2009). Differences between groups were evaluated using Student's unpaired *t*‐test.

All numerical values and *P*‐values are listed in the tables in the Appendix. In the text, *n* = number of cells, and mean values are quoted as the means ± SD. In all figures, the level of statistically significant difference is as follows: *P ≤ 0.05, **P ≤ 0.01 and ***P ≤ 0.001.

## 3 RESULTS

### 3.1 Patch‐clamp recordings

A voltage‐step protocol was used, which is best suited for describing the macroscopic currents expressed by type I and II hair cells, consisting of an initial holding potential (*V*_hold_) of −70 mV, followed by a hyperpolarizing step to −120 mV for 150 ms, then 10 mV steps between −70 and 40 mV for 500 ms, followed by a step to −40 mV for 200 ms and a final return to *V*_hold_ of −70 mV. Type I and type II hair cells were distinguished depending on the presence or absence of *I*_KL_ as illustrated below.

### 3.2 Effects of linopirdine on type II hair cells

**FIGURE 1:**
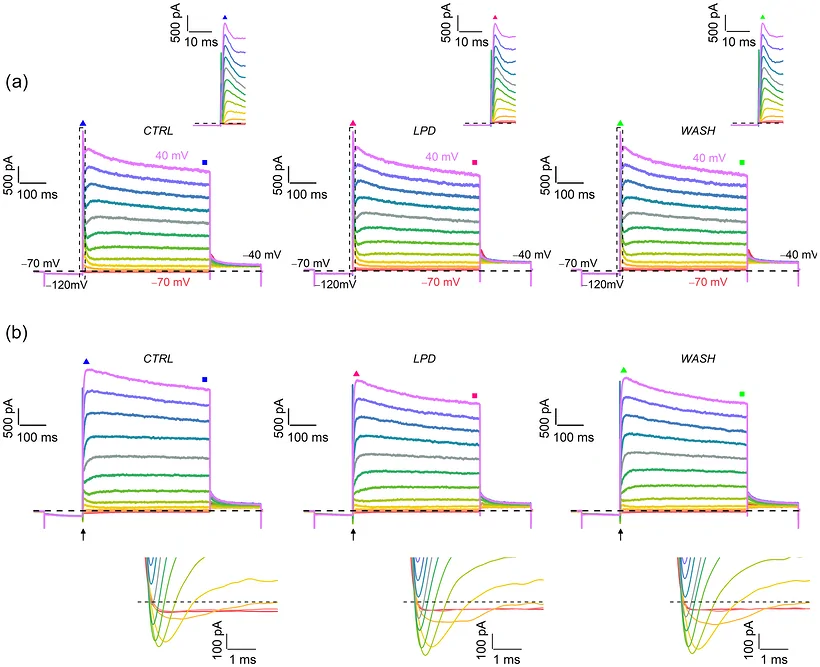
Effects of linopirdine on type II hair cells. (a) Representative current response from a type II hair cell (embryonic day 19), showing a large transient A‐type K^+^ current (*I*_KA_) in control conditions (CTRL; left panel), during perfusion of 10 µM linopirdine (LPD; central panel) and after wash‐out (WASH; right panel). Currents were elicited using the voltage protocol described in the text. The dashed rectangle frames *I*_KA_, which appeared immediately after the capacitive artifact. The corresponding expanded traces are shown above each panel. The filled triangles indicate the time point at which the peak current was measured, and the steady‐state current was measured towards the end of the depolarizing steps (filled squares). (b) Representative whole‐cell currents recorded from a type II hair cell (embryonic day 20) expressing a small *I*_KA,_ distinguishable only at less depolarized voltages, and a predominant delayed rectifier current (*I*_KV_) in control conditions (left panel), in the presence of 10 µM LPD (central panel) and after wash‐out (right panel). The expanded traces at the bottom show the Na^+^ current (*I*_Na_), which was detectable in this cell upon depolarization from −120 mV (arrow), on an expanded base time.

To assess the contribution of the K_V_7.4 α‐subunit (also known as KCNQ4) to the outward K^+^ current, we tested the effect of 10 µM linopirdine, a concentration at which it specifically blocks KCNQ2–KCNQ5 channels (Brown & Passmore, 2009). Initially, we examined the effect of linopirdine on the current responses recorded from type II hair cells. These cells showed variable combinations of transient (*I*_KA_) and sustained (*I*_KV_) outward K^+^ currents, as shown by the two representative examples in Figure 1.

**FIGURE 2:**
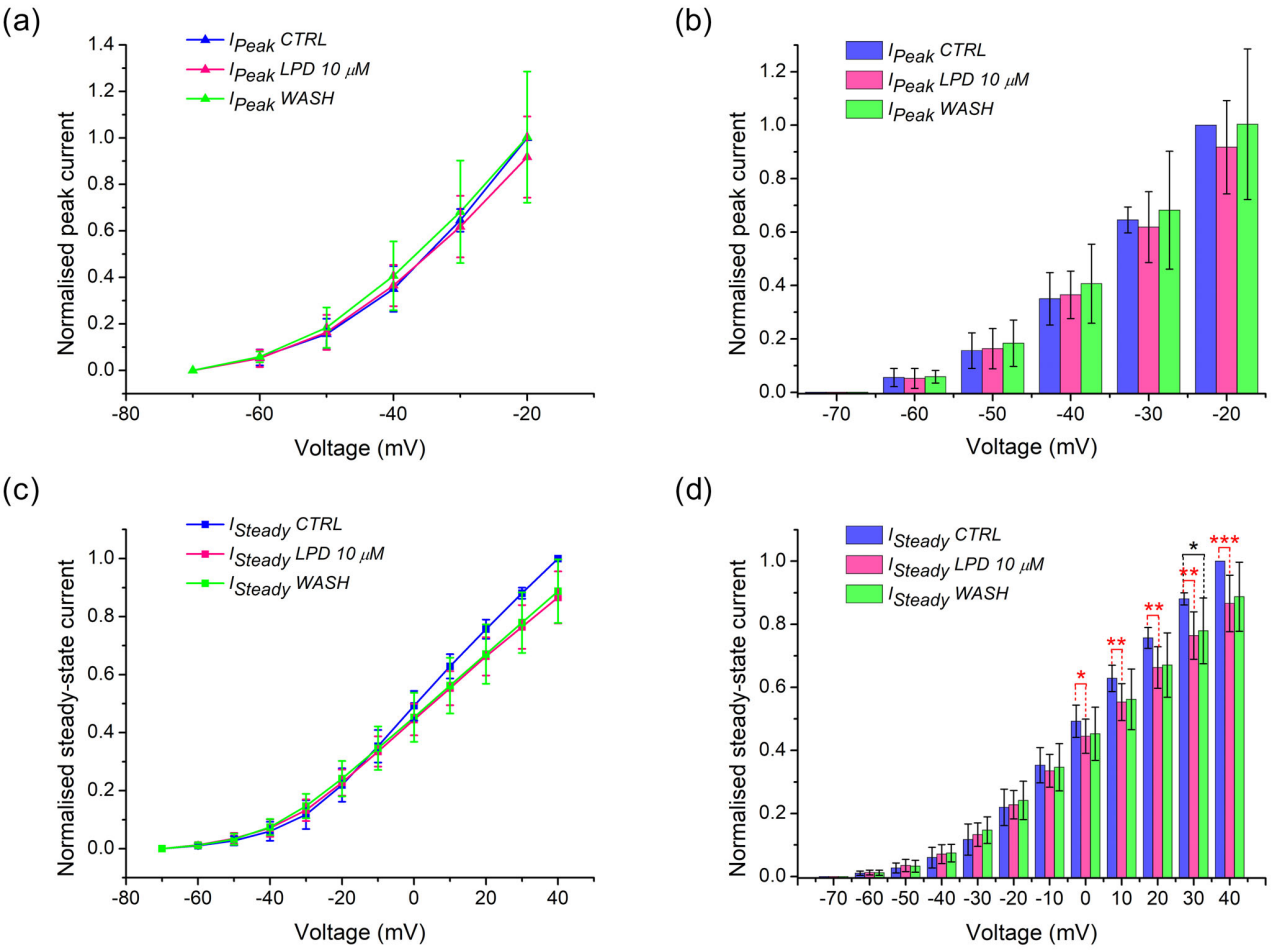
Average effects of linopirdine on peak and steady‐state K^+^ currents in type II hair cells. Average normalized current–voltage (*I–V*) relationship of the peak (a,b) and steady‐state (c,d) outward K^+^ current recorded from type II hair cells (*n* = 12; range embryonic day 15–21) in control conditions (blue), during application of 10 µM linopirdine (pink) and after wash‐out (green). (a,c) Averaged *I–V* curves. (b,d) Corresponding bar plots with statistical analysis. Linopirdine (10 µM) did not significantly affect peak current amplitudes (a,b), whereas it induced a significant reduction of the steady‐state current only at positive membrane potentials (c,d). Red asterisks indicate statistically significant differences between control and linopirdine conditions; black asterisks indicate statistically significant differences between control and wash‐out.

We found that, as in the examples shown in Figure 1 and despite the variable contribution of *I*_KA_ and *I*_KV_ to the macroscopic current recorded from different type II hair cells, linopirdine produced only a small reduction of the steady‐state outward current at most depolarized membrane potentials (≥0 mV). The average normalized current–voltage (*I*–*V*) relationships for peak and steady‐state outward currents are shown in Figure 2 (all numerical values are provided in the Appendix, Tables A1 and A2). Given that, at most depolarized voltages, the increasing speed of activation of *I*_KV_ makes it impossible to distinguish the peak of *I*_KA_, peak amplitude was measured only up to −20 mV (Figure 2a). Statistical analysis revealed no significant differences in peak current before and after perfusion with 10 µM linopirdine across the voltage range shown (Figure 2b). The steady‐state current, which was measured toward the end of the depolarizing voltage steps, was measured up to 40 mV (Figure 2c). Because *I*_KA_ inactivates rapidly (e.g., the inactivation time constant was 29 ms at −20 mV for the cell response shown in Figure 1a), the steady‐state current measured towards the end of the 500 ms at most depolarized voltage steps consisted solely of *I*_KV_. A small albeit significant reduction of the steady‐state current was seen after linopirdine at depolarized voltages starting from 0 mV (Figure 2d, red asterisks). However, following wash‐out of the drug, the outward current recovered only minimally, suggesting that most, if not all, of the observed reduction could be attributed to current rundown.

**FIGURE 3:**
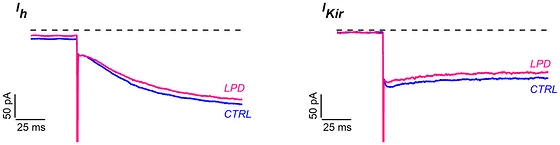
Effect of linopirdine on inward rectifying currents in type II hair cells. Representative inward currents recorded from two type II hair cells in response to a hyperpolarizing step at −120 mV from *V*_hold_ of −70 mV. The left panel shows a cell expressing the mixed Na^+^/K^+^ inward current, *I*_h_. The right panel shows a cell expressing the inwardly rectifying K^+^ current, *I*_Kir_. For both cells, inward currents were recorded in control conditions (CTRL) and during application of 100 µM linopirdine. No effects of linopirdine were detectable.

The presence of the inward rectifying mixed Na^+^/K^+^ currents (*I*_h_) and of the inward (anomalous) rectifying K^+^ current (*I*_Kir_) was assessed by analysing the current responses elicited by a hyperpolarizing step to −120 mV from *V*_hold_ of −70 mV (Figure 3). Although linopirdine is considered a specific blocker of K_V_7 channels in mammalian cells, we nevertheless tested this drug at concentrations of 10 and 100 µM on inward rectifying currents expressed by chicken embryo type II hair cells. No effect was found at either concentration; therefore, data were pooled. In *I*_h_‐expressing cells, the average steady‐state current showed a slight, non‐significant reduction (−191.19 ± 110 pA in control conditions vs. −165.25 ± 98 pA in linopirdine; *n* = 8; *W* = 24, *P* = 0.1094, Wilcoxon matched‐pairs signed rank test). In *I*_Kir_‐expressing cells, neither the average peak current (−204.96 ± 53 pA, vs. −185.72 ± 39 pA in linopirdine; *n* = 5; *t* = 1.918, d.f. = 4, *P* = 0.1276, Student's paired *t*‐test) nor the average steady‐state current (−194.15 ± 47 vs. −173.90 ± 37 pA; *n* = 5; *t* = 2.437, d.f. = 4, *P* = 0.0715, Student's paired *t*‐test) was significantly affected.

### 3.3 Effects of linopirdine on type I hair cells

**FIGURE 4:**
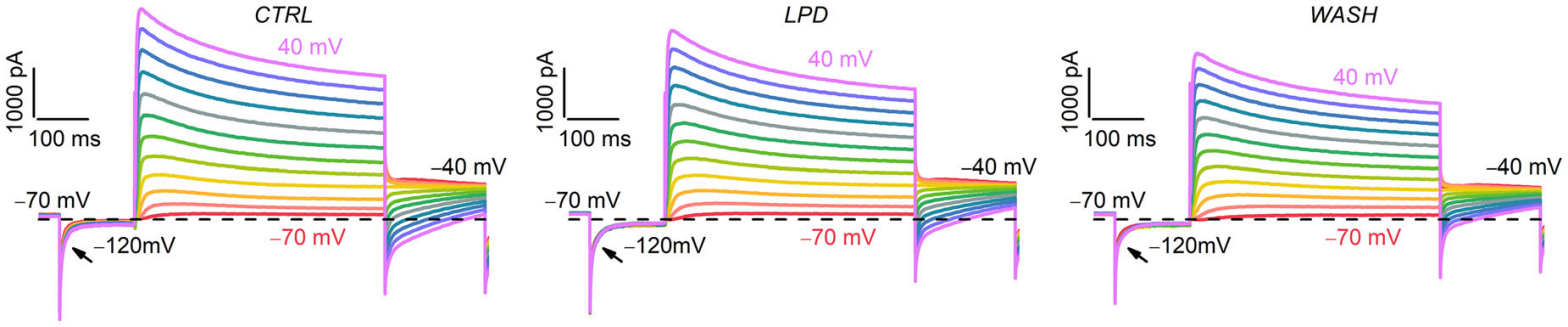
Effects of linopirdine on type I hair cells. Representative current responses recorded from a type I hair cell (embryonic day 18) in control conditions (CTRL; left panel), during perfusion of 10 µM linopirdine (LPD; central panel) and after wash‐out (WASH; right panel). Currents were elicited using the same voltage protocol as for type II hair cells and described at the beginning of the Results section. The arrow indicates the *I*_KL_ deactivation time course. Note that *I*_KL_ deactivates completely during the 150 ms hyperpolarizing step of −120 mV. Also note that the outward K^+^ current elicited by voltage steps between −70 and 40 mV shows increasing relaxation with increasing depolarization and that the instantaneous tail current upon repolarization to −40 mV is outward following steps between −70 and 0 mV, but then reverses, and the amplitude of the inward tail current increases following increasing depolarizing steps. The above results are consistent with K^+^ accumulation in the residual calyceal synaptic cleft (see Materials and methods).

The effect of 10 µM linopirdine was also assessed in type I hair cells, identified by the presence of *I*_KL_, which is largely active at a *V*_hold_ of −70 mV and therefore shows a pronounced deactivation when the cell is hyperpolarized to −120 mV from *V*_hold_. Representative current responses are shown in Figure 4. Linopirdine did not produce clear changes in the macroscopic current amplitude or kinetics of *I*_KL_.

**FIGURE 5:**
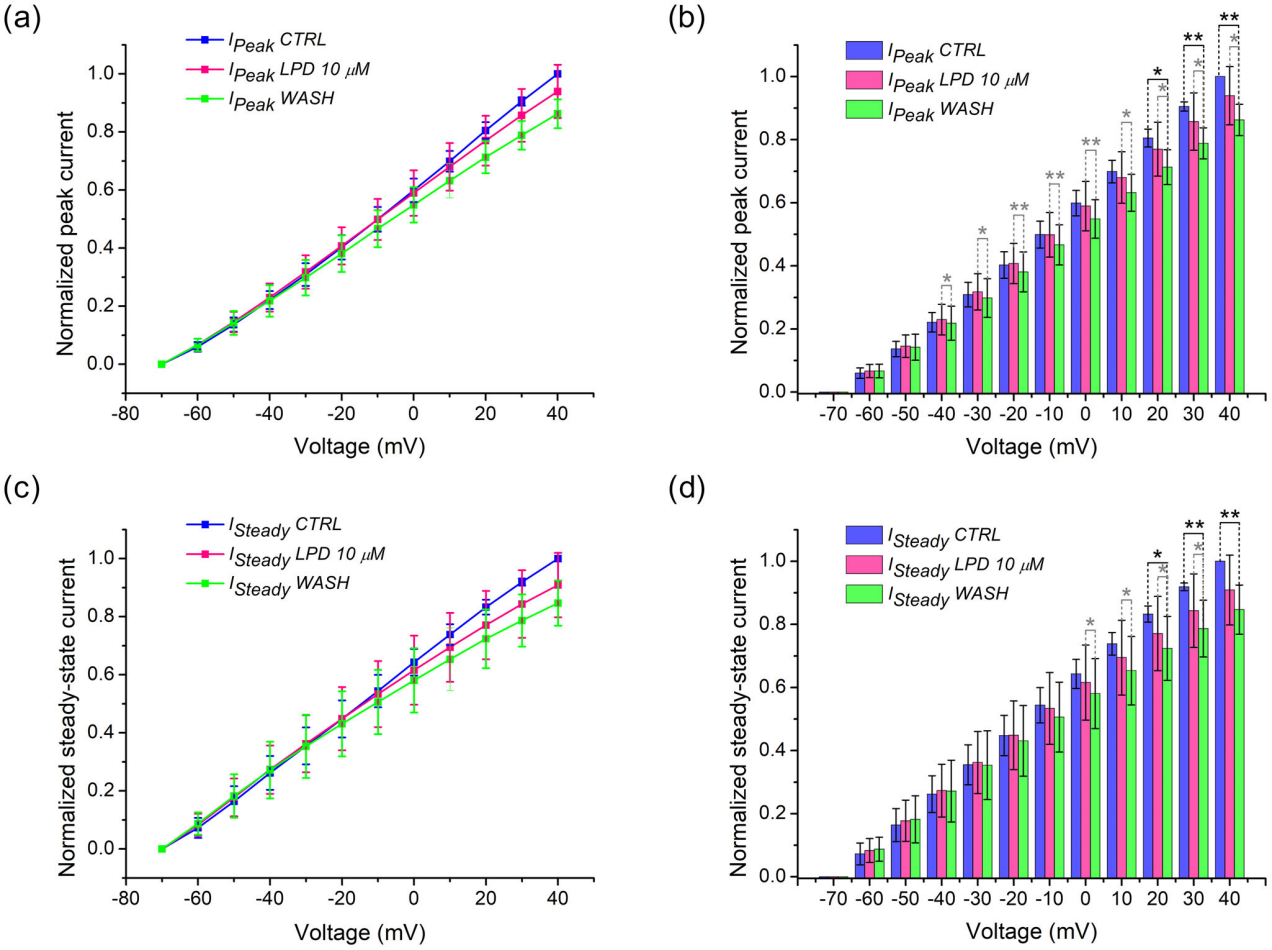
Average effects of linopirdine on peak and steady‐state K^+^ currents in type I hair cells. Average normalized current–voltage (*I–V*) relationship of the peak (a,b) and steady‐state (c,d) K^+^ currents recorded from type I hair cells (*n* = 8; range embryonic day 17–21) in control conditions (blue), during application of 10 µM linopirdine (pink) and after wash‐out (green). (a,c) Averaged *I–V* curves. (b,d) Corresponding bar plots with statistical analysis. A small reduction in both peak and steady‐state current amplitudes was observed at more depolarized membrane potentials during linopirdine perfusion and after wash‐out. Statistical analysis revealed significant differences between linopirdine and wash‐out (grey asterisks), and between control and wash‐out, at selected voltages (black asterisks).

The average normalized peak and steady‐state *I*–*V* relationships are shown in Figure 5 (all numerical values are provided in the Appendix, Tables A3 and A4). A slight reduction in current amplitude was observed at most depolarized membrane potentials during linopirdine perfusion, which, however, continued during wash‐out. Statistical analysis revealed significant differences between linopirdine and wash‐out (grey asterisks), and between control and wash‐out (black asterisks), at strongly depolarized potentials. The progressive decline in current amplitude across experimental conditions, together with the absence of a selective or reversible effect during drug application, indicates that the observed reduction is attributable to current rundown rather than a specific pharmacological action of linopirdine. This interpretation is consistent with the previously reported dependence of *I*_KL_ upon diffusible factors that are dialysed by the patch pipette solution in ruptured whole‐cell configuration (Hurley et al., 2006). We will see that this explanation is also consistent with the result described in the next section.

In summary, patch‐clamp recordings indicate that K_V_7 channels do not contribute to the macroscopic K^+^ current recorded from chicken embryo type I and II hair cells.

### 3.4 Messenger RNA expression of K^+^ channels in vestibular semicircular canals from E16 and E20 chicken embryos; RT‐PCR

**FIGURE 6:**
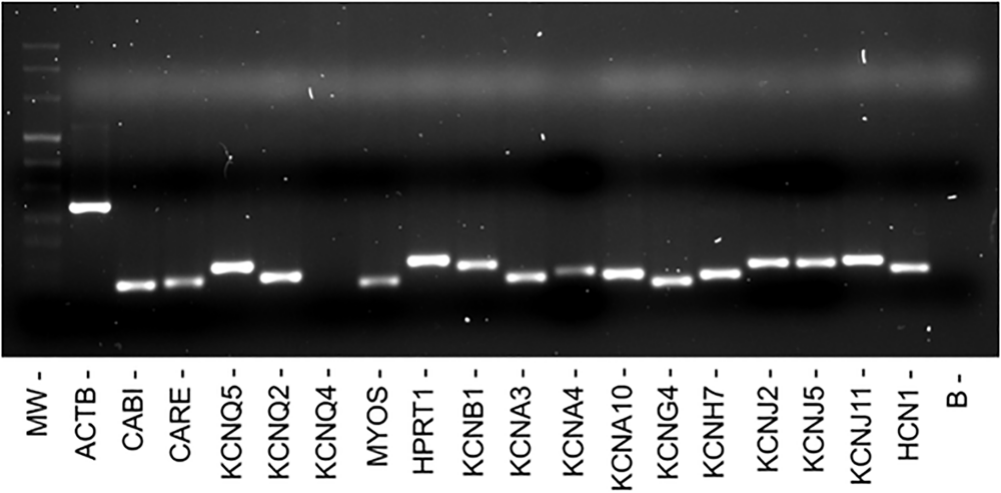
Agarose gel electrophoresis of PCR products. PCR amplicons for the target genes (as labelled in the figure; see Table 1) were separated on an agarose gel, stained with ethidium bromide and visualized using the iBright™ CL1000 Imaging System. The molecular weight marker (MW) was used as a size reference. Samples loaded correspond to the embryonic day 20 chicken embryo developmental stage (similar results were also obtained with the embryonic day 16 samples; not shown). Each lane represents an individual sample. All PCR products are of the expected size, with no evidence of non‐specific amplification or primer‐dimers. The expected amplicon sizes for each gene are reported in Table 1. B, negative control.

**FIGURE 7:**
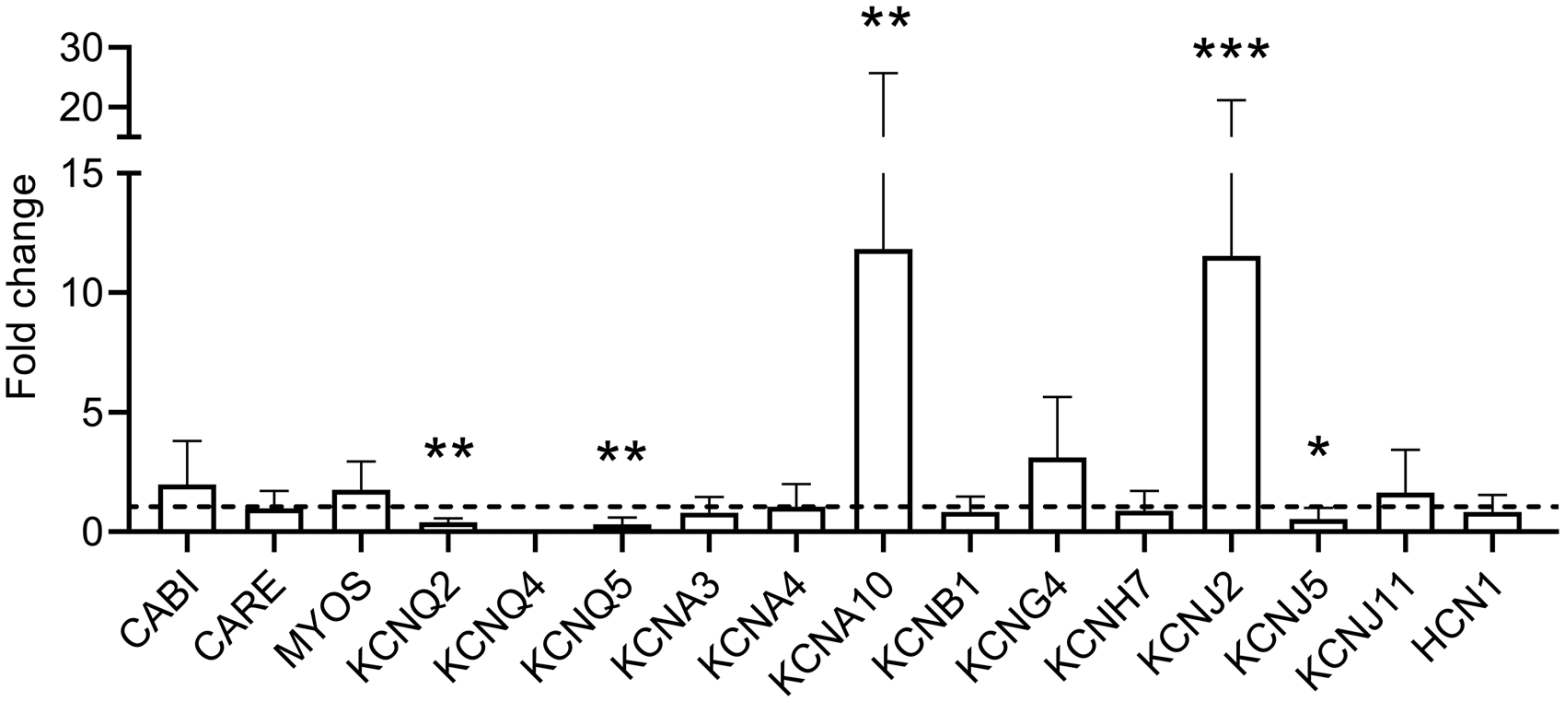
Relative gene expression levels of K^+^ channel genes at embryonic day (E)20 relative to E16. Fold changes were calculated using the ΔΔCt method, with expression levels normalized to housekeeping genes and referenced to the E16 stage (baseline = 1, dashed line). See Materials and Methods for details. Bars represent the mean ± SD from four independent experiments (*n* = 4). Statistical significance was determined using Student's unpaired *t*‐test, with genes marked by the asterisk showing significant differences compared with E16 (*P* < 0.05).

K_V_7.4 channels appear to contribute to *I*_KL_ in mouse immature type I hair cells (Holt et al., 2007) and to *I*_KV_ in mouse mature type I and II hair cells (Martin et al., 2024). However, we found linopirdine to be ineffective when perfused to developing type I and II hair cells. To gain more insight into the channels contributing to K^+^ currents in differentiating type I and II hair cells, we investigated mRNA expression of K^+^ channels previously reported in chick (P7; Scheibinger et al., 2022] and/or rodent (Wang et al., 2024) vestibular sensory epithelia, by qPCR analysis at E16 and E20. Gel electrophoresis (Figure 6) showed that several K^+^ channel subunits were expressed in the vestibular organ at both embryonic ages, including K_V_1.8 channels, which in mouse mature type I hair cells sustain *I*_KL_ and in mature type II hair cells contribute to *I*_KA_ (Martin et al., 2024), but not K_V_7.4. Additional qPCR analysis revealed a downregulation of K_V_7.2 (*Kcnq2*), K_V_7.5 (*Kcnq5*) and K_ir_3.4 (*Kcnj5*) between E16 and E20, which decreased by ∼60%, ∼70% and ∼50%, respectively (Figure 7; numerical values are provided in the Appendix, Table A5). By contrast, K_V_1.8 (*Kcna10*) and K_ir_2.1 (*Kcnj2*) were upregulated (Figure 7).

Thus, RT‐PCR experiments confirmed the absence of K_V_7.4, while suggesting the possibility that *I*_KL_ in chicken embryo type I hair cells consists of K_V_1.8, as in mature rodents. Indeed, the 10‐fold increase in expression of *Kcna10* between E16 and E20 appears consistent with the functional differentiation of type I hair cells based on the expression of *I*_KL_ from E17 (Masetto et al., 2000). Moreover, the 10‐fold increase of *Kcnj2* expression is consistent with the final maturation of type II hair cells by acquiring the inward rectifying K^+^ current from E19 (Masetto et al., 2000). Finally, RT‐PCR experiments suggest the possible contribution of several other K^+^ channel subunits to the K^+^ currents recorded from type I and II hair cells. To clarify this aspect, we performed Patch‐seq experiments.

### 3.5 Patch‐seq experiments on type I and type II hair cells

The Patch‐seq technique can provide the complete transcriptome of a single cell following patch‐clamp recording from the same cell. Until now, such data had not been obtained from hair cells.

To minimize the time of recording for Patch‐seq experiments (see Materials and methods), a simplified voltage protocol was delivered before aspirating the hair cell cytoplasm, which allowed identification of the hair cell type. The voltage‐clamp protocol consisted of a *V*_hold_ of −70 mV, a hyperpolarizing step to −120 mV for 300 ms, then four or six steps from −100 mV in 20 mV increments for 500 ms, a step to −40 mV for 200 ms, and final return to *V*_hold_ of −70 mV. Type I and type II hair cells were distinguished depending on the presence or absence of *I*_KL_, respectively.

**FIGURE 8:**
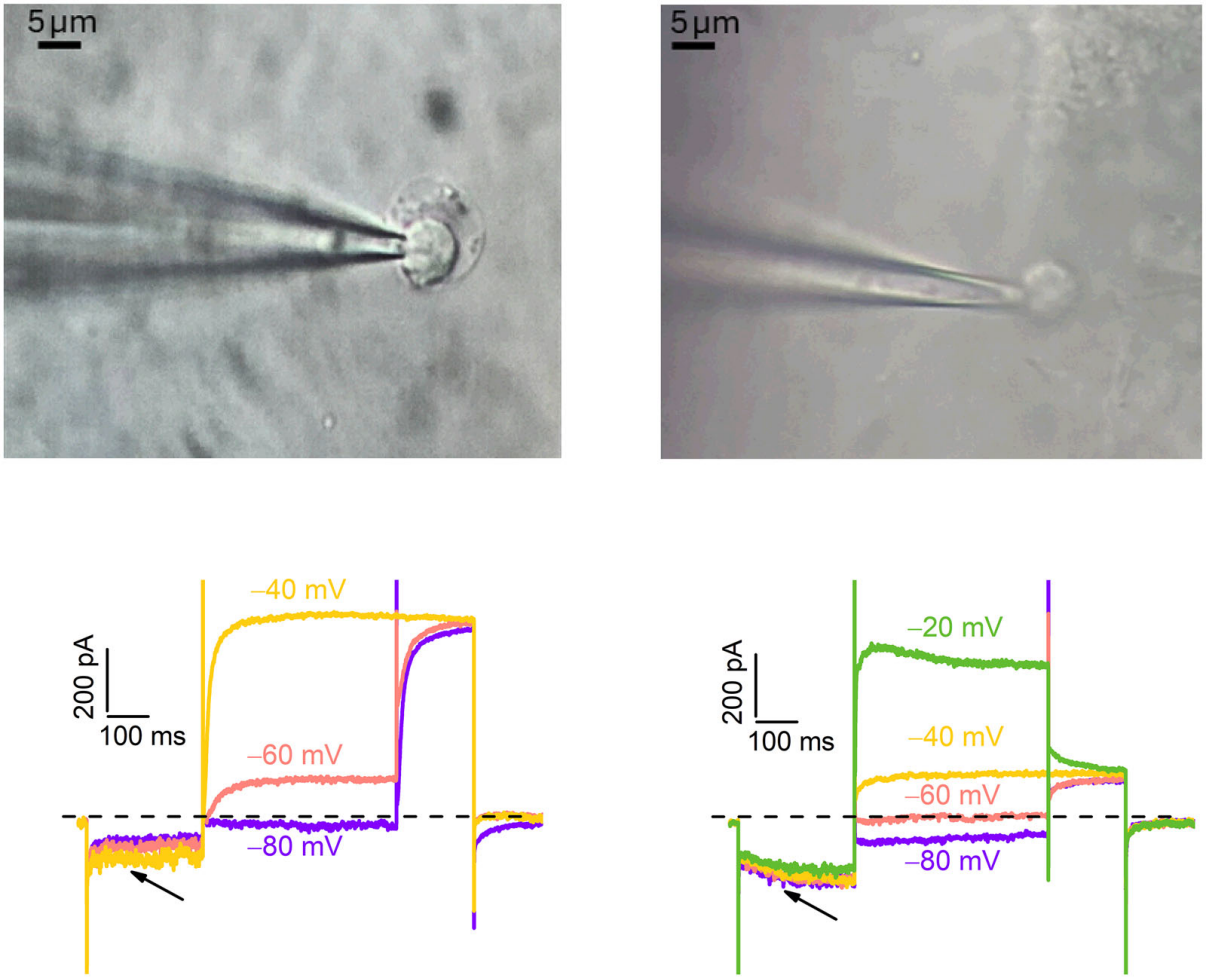
Patch‐sequencing recordings. Top panels, photomicrographs showing a type I hair cell (left; embryonic day 20) and a type II hair cell (right; embryonic day 19) attached to the tip of the patch pipette, after whole‐cell recording, cytoplasm aspiration and retraction from the sensory epithelium. Bottom panels, current responses recorded from the cells shown above. Note that in the type I hair cell (bottom left) a fast‐decaying current (arrow) was present upon hyperpolarization from *V*_hold_ of −70 mV to −120 mV, which can be explained by the deactivation of the fraction of *I*_KL_ active at *V*_hold_. Moreover, a significant outward K^+^ current could be elicited upon depolarization from −120 to −60 mV (orange trace). This current response is consistent with the expression of the low‐voltage‐activated outward rectifying K^+^ current, *I*_KL_. In the type II hair cell (bottom right), in contrast, no outward K^+^ current was elicited upon depolarization to −60 mV from −120 mV (orange trace), consistent with the lack of *I*_KL_. An additional step is shown for the type II hair cell, corresponding to the current response elicited by depolarization to −20 mV (green trace), to show the *I*_KV_ time course better. Finally, a slowly activating inward current can be seen in the type II hair cell upon hyperpolarization from a *V*_hold_ of −70 mV to −120 mV (arrow), which can be identified as the mixed Na^+^/K^+^ inward rectifying current, *I*_h_.

We were able to obtain viable cDNA from four whole‐cell‐recorded hair cells, although the highest score (see e.g., Lipovsek et al., 2020, 2021) was reached only for an E20 type I and an E19 type II hair cell (see Materials and Methods). The relevant photomicrographs and current responses are shown in Figure 8.

We found 2300 and 2336 genes expressed by the type I and the type II hair cell, respectively; of these, 1874 genes were common to both cell types. Consequently, it was found that 426 genes were expressed exclusively by the type I hair cell and another 462 genes by the type II hair cell. Sequencing revealed the absence of the K_V_7.4 subunit in both hair cell types, a finding consistent with results obtained using linopirdine and with RT‐PCR analysis.

Further analysis of Patch‐seq data revealed the presence of several voltage‐gated ion channel transcripts in both hair cells, which are listed below and discussed in detail in the Discussion section.

Concerning the delayed rectifier class of K^+^ channels, we found transcripts for the K_V_1.8 (*Kcna10*), the K_V_1.6 (*Kcna6*) and the K_V_4.2 (*Kcnd2*) α‐subunits. We also found transcripts for the K_Ca_1.1 (*Kcnma1*) α‐subunit, which mediates the large conductance (BK) Ca^2+^‐dependent outward K^+^ current; the Na_V_1.7 (*Scn9a*) α‐subunit, which can account for *I*_Na_ found in most hair cells of the chicken embryo (Figure 1; Giunta et al., 2024; Masetto et al., 2000; 2003), the Ca_V_1.3 (*Cacna1D*) α‐subunit, accounting for *I*_Ca_ found in all hair cells, and the K_ir_2.2 (*Kcnj12*) and K_ir_5.1 (*Kcnj16*) α‐subunits, which sustain the anomalous inward rectifying K^+^ current (*I*_Kir_). However, the presence of *I*_Kir_ is unclear in the current responses shown in Figure 8, probably because its amplitude was too small. In contrast, despite the presence of the inward rectifying mixed Na^+^/K^+^ current, *I*_h_, in the response of the type II hair cell shown in Figure 8 (bottom right panel), we did not find the transcript for *Hcn* channels, presumably because it was below the detection threshold.

The transcripts for voltage‐gated ion channel subunits are listed in Table 2.

**TABLE 2:** Transcripts for voltage‐gated ion channels in type I and type II hair cells.

Gene	Protein	Type I	Type II
*Cacna1D*	CaV1.3	✔	✔
*Kcna6*	KV1.6	✔	✔
*Kcna10*	KV1.8	✔	✔
*Kcnd2*	KV4.2	✔	✔
*Kcnj12*	Kir2.2	✔	✔
*Kcnj16*	Kir5.1	✔	✔
*Kcnma1*	KCa1.1	✔	✔
*Kcnq2*	KV7.2	✔	
*Scn3b*	Voltage‐gated sodium channel β3 subunit (Navβ3)	✔	✔
*Scn9a*	NaV1.7	✔	✔

Regarding non‐voltage‐gated ion channels, the transcripts of greatest relevance to hair cells are listed in Table 3 and discussed in the Discussion section.

**TABLE 3:** Other relevant transcripts in type I and type II hair cells.

Gene	Protein	Type I	Type II
*Agr3*	Anterior Gradient Protein 3 homolog	✔	✔
*Cfap126*	*CFAP126*	✔	✔
*Chrna10*	α10 nicotinic acetylcholine receptor subunit	✔	
*Chrna9*	α9 nicotinic acetylcholine receptor subunit	✔	✔
*Cib3*	CIB3	✔	✔
*Cluap1*	*CLUAP1*	✔	✔
Coch	Cochlin	✔	✔
*Ctbp1*	C‐terminal‐binding protein 1	✔	✔
*Ctbp2*	C‐terminal‐binding protein 2	✔	
*Fat4*	FAT atypical cadherin 4 (or FAT4 cadherin)		✔
*Gjb6*	Gap junction beta‐6 protein	✔	✔
*Lfng*	β1,3‐*N*‐acetylglucosaminyltransferase)	✔	
*Myo1d*	Myosin‐Id	✔	✔
*Myo1h*	Myosin‐Ih	✔	✔
Myo6	Myosin VI	✔	✔
*Ocm1*	Oncomodulin	✔	✔
*Otof*	Otoferlin	✔	
*Pcdh20*	Protocadherin‐20	✔	✔
*Pclo*	*Piccolo*	✔	✔
*Snap‐25*	Synaptosomal‐associated protein 25		✔
*Sox2*	SRY‐box transcription factor 2	✔	✔
*Spp1*	Osteopontin	✔	
*Stard10*	StAR‐related lipid transfer protein 10	✔	✔
*Tmc1*	TMC1	✔	✔

### 3.6 Whole‐organ transcriptome

The RT‐PCR experiments were targeted to a few representative ion channel subunits. However, following Patch‐seq analysis, we were surprised to find no transcripts for *Myo7A* (Myosin 7A), a key protein involved in organization of the actin cytoskeleton that is considered a marker of mature hair cells (McInturff et al., 2018; Scheibinger et al., 2022; Xu et al., 2025). Therefore, we also performed whole‐organ transcriptome experiments from 22 chicken embryos at E16. In addition to confirming the presence of all mRNAs detected by RT‐PCR at E16 and at E20, whole‐organ transcriptome analysis also revealed the presence of the Myosin 7A transcript at E16, indicating that the amount obtained from Patch‐seq experiments at E19 and E20 was below the detection threshold.

## 4 DISCUSSION

In this study, we used a multi‐omics approach to gain insight into the molecular nature of ion channels expressed by type I and II hair cells in the chicken embryo. We identified ion channel subunits also expressed in mammalian hair cells, in addition to new subunits not previously reported in these cells. This study provides the first transcript database of identified single type I and II hair cells, which will prove useful for comparative studies.

### 4.1 Voltage‐gated K^+^ channels

In patch‐clamp experiments, type I and type II hair cells are distinguished by the expression of the low‐voltage‐activated outward rectifying K^+^ current termed *I*_KL_. In chicken embryo hair cells, *I*_KL_ first appears at E17, expressed together with a delayed rectifying K^+^ current (*I*_KV_), which activates 40 mV more depolarized (−50 vs. −90 mV; Masetto et al., 2000). In mouse vestibular hair cells, *I*_KL_ is expressed from E18 (Géléoc et al., 2004), although in rats it appears only after birth (Hurley et al., 2006; Rüsch et al., 1998). The K_V_7.4 channel α‐subunit (*Kcnq4*) was initially proposed to carry *I*_KL_ in neonatal mouse type I hair cells (Holt et al., 2007). In adult mice, however, *I*_KL_ is normally present in *Kcnq4* ^−/−^ mice (Spitzmaul et al., 2013), and K_V_7.4 expression appears to be confined to the afferent nerve calyx. In contrast, *I*_KL_ is absent in adult *Kcna10* ^−/−^ mice, which lack the K_V_1.8 α‐subunit (Martin et al., 2024). It is therefore possible that the K_V_7.4 channel α‐subunit is expressed in mouse type I hair cells during development but disappears as the animal matures, remaining only in the postsynaptic calyx (Hong et al., 2024; Spitzmaul et al., 2013).

In this study, we demonstrated that the K_V_7.4 subunit is not present in immature or mature type I and type II hair cells. Patch‐seq experiments additionally showed that, as in mice, the K_V_1.8 α‐subunit is expressed in type I and type II hair cells (E19–E20). Whether *I*_KL_ in the chicken embryo type I hair cell is carried by homotetrameric K_V_1.8 channels, as proposed in adult mice (Martin et al., 2024), remains to be clarified. Indeed, our Patch‐seq experiments showed the additional expression of K_V_1.6 in type I and type II hair cells, which could co‐assemble with K_V_1.8. Therefore, the *I*_KL_ recorded in this study could arise from homotetrameric K_V_1.8 channels and/or heteromeric K_V_1.6 and K_V_1.8 channels, although no evidence of a K_V_1.6/K_V_1.8 heterotetramer has been reported to date. Homotetrameric K_V_1.6 channels, in contrast, exhibit rapid activation and slow inactivation kinetics (Cai & Sesti, 2007). Given that *I*_KL_ does not undergo inactivation, if the K_V_1.6 α‐subunit assembles to form a homotetrameric channel in type I and II hair cells, it would be expected to carry a slowly inactivating K^+^ current (i.e., *I*_KV_). Indeed, a distinctive feature of the K_V_1.6 subunit is the presence of an N‐type inactivation prevention (NIP) domain at the N‐terminus (Cai & Sesti, 2007). This domain prevents rapid (N‐type) inactivation induced by auxiliary subunits, such as Kvβ1 (*Kcnab1*), which we found to be expressed in both hair cell types. The K_V_1.6 subunit has been reported in mouse crista hair cells (Wilkerson et al., 2021).

In contrast, because K_V_1.8 channels lack the NIP domain, those associated with the auxiliary subunit Kvβ1 would produce a rapidly inactivating outward rectifying K^+^ current. This could explain why mice lacking K_V_1.8, besides missing the sustained *I*_KL_ in type I hair cells, also lacked the rapidly inactivating A‐type K^+^ current in type II hair cells (Martin et al., 2024). In other words, in type I hair cells the K_V_1.8 subunit could form homotetrameric channels to generate *I*_KL_, and in type II hair cells it could associate with K_V_β1 to produce *I*_KA_. K_V_β1 is present in mouse type II hair cells (McInturff et al., 2018). However, it should be noted that the K_V_1.4 α‐subunit is also expressed in mouse type II but not type I hair cells (McInturff et al., 2018), which could contribute to *I*_KA_. It is possible that its transcript has escaped detection by the Patch‐seq approach.

The K_V_4.2 and K_Ca_1.1 α‐subunits were also identified, for the first time, in both hair cell types. K_V_4.2 (*Kcnd2*) mediates a rapidly inactivating, A‐type outward K^+^ current that is not under the control of the N‐terminus as it is in Shaker channels (Gutman et al., 2005; Zhu et al., 1999). K_V_4 subunits can form heterotetrameric channels with other members of the same subfamily (Shals) of delayed rectifiers, but these were not detected in the present study. It is therefore possible that, in vestibular hair cells, K_V_4.2 subunits form homotetrameric channels and might contribute to the *I*_KA_ recorded in type II hair cells. The role of K_V_4.2 in type I hair cells, however, remains unclear, because these do not express rapidly inactivating K^+^ currents. Transcripts of the auxiliary subunit *Kcnip4* (potassium voltage‐gated channel interacting protein 4) have also been found in both hair cell types. This protein is an integral subunit of native K_V_4 channel complexes, which can regulate A‐type K^+^ currents in response to changes in intracellular Ca^2+^ (Han et al., 2006). Interestingly, K^+^ current inactivation varies significantly depending on the position of the hair cell in the sensory crista, showing a decreasing rate and importance from the periphery towards the centre (Masetto & Correia, 1997). This scenario could be explained by the different amounts and combinations of α and auxiliary K^+^ channel subunits expressed by hair cells in different regions of the crista.

K_Ca_1.1 (*Kcnma1*) is the α‐subunit of the BK‐type (big conductance) Ca^2+^‐activated K^+^ channel. A Ca^2+^‐dependent K^+^ current has been reported in vestibular hair cells of chicken embryos from E14 onwards, although its molecular nature has not been identified (Masetto et al., 2000). In mice, its expression has been reported to be restricted to striolar type I hair cells (McInturff et al., 2018).

Kir2.2 (*Kcnj12*) and Kir5.1 (*Kcnj16*) were found in both hair cell types, accounting for the (anomalous) inward rectifying K^+^ current (*I*_Kir_). *I*_Kir_ was previously found in chicken embryo vestibular hair cells from E19, being the last ionic current to appear (Masetto et al., 2000).

### 4.2 Other voltage‐gated ion channels

The Ca_V_1.3 α‐subunit (*Cacna1d*) was the only Ca^2+^ channel α‐subunit found in this study and would therefore be responsible for the entire Ca^2+^ current previously recorded in chicken embryo type I and II hair cells from E10 onwards (Masetto et al., 2000; 2005). The Ca_V_1.3 α‐subunit is also primarily responsible for *I*_Ca_ in rodent vestibular hair cells (Manca et al., 2021; Spaiardi et al., 2022). In mice, additional Ca^2+^ channel subunits have been hypothesized based on residual *I*_Ca_ in Ca_V_1.3^−/−^ mice (Dou et al., 2004), although they have never been demonstrated or identified. However, synaptic vesicle exocytosis was absent in type I and II vestibular hair cells of mature Ca_V_1.3^−/−^ mice, indicating that non‐Ca_V_1.3 channels are not directly involved in signal transmission (Spaiardi et al., 2022). Given that analysis of *I*_Ca_ in wild‐type type I and II hair cells has never revealed the presence of an additional component of *I*_Ca_, the question arises whether non‐Ca_V_1.3 channel subunits might be expressed only in Ca_V_1.3^−/−^ mice to compensate for the deficiency. That said, it remains possible that also for Ca^2+^ channels, transcripts for other subunits simply went undetected.

Na_V_1.7 (*Scn9a*) was the only Na^+^ channel α‐subunit transcript found in this study and should therefore account for at least some of the *I*_Na_ found in embryonic and adult chicken vestibular hair cells (Masetto et al., 2000; 2003). The absence of *I*_Na_ in the two hair cells sequenced (Figure 8c,d) despite the presence of the transcript suggests a low functional expression of the protein in these cells. In rodents, *I*_Na_ has been found only in immature vestibular hair cells (Chabbert et al., 2003; Géléoc et al., 2004; Lennan et al., 1999; Wooltorton et al., 2007), and its molecular nature is unknown. In both birds and rodents, *I*_Na_ recorded from vestibular hair cells displays the peculiar property of a hyperpolarized inactivation curve, such that most sodium channels are unavailable for depolarization from the resting membrane potential of the cell. In other words, hair cells must be strongly hyperpolarized (apparently non‐physiologically) to recruit *I*_Na_. No spontaneous action potential activity has ever been recorded in vestibular hair cells. In immature cochlear inner hair cells, in contrast, *I*_Na_ contributes to Ca^2+^ action potentials (Eckrich et al., 2012). In inner hair cells, however, *I*_Na_ displays a less negatively shifted inactivation curve, and labelling by antibodies directed against Na_V_1.1 and Na_V_1.6 subunits was detected (Eckrich et al., 2012). We also found the transcript for the voltage‐gated Na^+^ channel β‐subunit 3 (*Scn3b*) in both hair cell types. This accessory protein modulates the activity of Na_V_1.2, causing a hyperpolarizing shift in the voltage dependence of inactivation of the channel (Morgan et al., 2000). Perhaps a similar function is also exerted on Na_V_1.7, because the auxiliary β‐subunit 3 has been reported to form a complex with Na_V_1.7 (Kanellopoulos et al., 2018).

### 4.3 Other relevant transcripts found in both hair cell types

As listed alphabetically in Table 3, in both hair cell types we found the transcripts for:

*Ctbp1* and *Ctpb2* (C‐Terminal Binding Protein 1 and 2), which localize to the ribbon synapse of hair cells (Uthaiah & Hudspeth, 2010). Both transcripts were also found in chick P7 hair cells (Schebinger et al., 2022), whereas only the transcript for *Ctbp2* was found in mouse hair cells (Xu et al., 2025).

*Cfap126*, a kinocilium protein, previously found in mouse vestibular hair cells (Xu et al., 2025).

*Chrna9* (Neuronal acetylcholine receptor subunit alpha‐9), previously found to be expressed in all P7 chick vestibular hair cells (Scheibinger et al., 2022) and in mouse vestibular type II hair cells (Poppi et al., 2023).

*Cib3* (Calcium and integrin binding family member 3), an auxiliary subunit of the mechanotransducer channel (Wang et al., 2023). *Cib3* transcripts are highly expressed in the extrastriolar region of the vestibular maculae. According to Wang et al. (2023), *Cib2* and *Cib3* transcripts have complementary expression patterns in the vestibular maculae, and they play different roles in stereocilia maintenance in vestibular hair cells. *Cib2* transcripts are highly expressed in the striolar region. The transcript for *Cib2* was not found here.

*Cluap1* (Clusterin associated protein 1), which is required for biogenesis of cilia. In mice, immunostaining confirmed the expression of *Cluap1* in the kinocilia of mature vestibular hair cells (Xu et al., 2025).

*Coch* (Cochlin), whose role is not fully understood, but mutations are linked to hearing loss and vestibular symptoms (DFNA9 dominantly inherited hearing impairment and vestibular dysfunction). In mice, *Coch* expression could not be assigned to a specific cell type (Xu et al., 2025).

*Myo1d* and *Myo1h*, myosins that regulate interactions between the plasma membrane and the actin cytoskeleton and contribute to vesicle trafficking. *Myo1h* expression has also been reported in mouse vestibular hair cells (Xu et al., 2025).

*Myo6* (Myosin VI), a protein enriched in the apical region of hair cells. *Myo6* expression has been reported in adult mouse type I and II hair cells (Xu et al., 2025). The absence of functional myosin VI has been associated with degeneration of hair bundles and impaired auditory or vestibular function (Hertzano et al., 2008).

*Ocm1* (Oncomodulin), a Ca^2+^‐binding protein. *Ocm* was found to be expressed by striolar hair cells (Scheibinger et al., 2022). In mice, *Ocm* is a known marker of striolar type I hair cells (McInturff et al., 2018).

*Pclo* (Piccolo), which is part of the presynaptic cytoskeletal matrix. It is expressed by chicken P7 hair cells (Schebinger et al., 2022), together with *Cbpt1* and *Cbpt2*. In mice, this transcript was found in all vestibular (and auditory) hair cells (Xu et al., 2025).

*Pcdh20* (Protocadherin‐20), a potential target for Pou2f3, a transcription factor critical for the formation of tuft cells, which are structurally similar to hair cells. In mice, it is expressed by type I and II hair cells (Xu et al., 2025).

*Sox2*, which encodes a member of the SRY‐related HMG‐box (*Sox*) family of transcription factors involved in the regulation of embryonic development, tissue formation and the determination of cell fate. In mice, *Sox2* is expressed by mature type II hair cells and by supporting cells (Jan et al., 2021; Wilkerson et al., 2021; Xu et al., 2025). In P7 chick utricles, *Sox2* labels type II hair cells located in the extrastriolar and striolar regions (Scheibinger et al., 2022).

*Stard10*, which encodes for a phospholipid transfer protein. It is a marker of P7 chick vestibular hair cells (Scheibinger et al., 2022).

*Tmc1* and *Tmc2* (Transmembrane channel‐like 1 and 2), which act as mechanically gated cation channels that convert hair bundle deflection into bioelectrical signals (Giese et al., 2025; Jia et al., 2020; Pan et al., 2018). These transcripts were previously found in P7 chick utricle hair cells, although *Tmc2* was found only in type I and II hair cells located in the striola (Scheibinger et al., 2022).

### 4.4 Type I vs. type II hair cells

In patch‐clamp experiments, type I hair cells are distinguished from type II hair cells by the expression of *I*_KL_. We found the same K^+^ channel subunits in the two hair cell types, with the exception of the K_V_7.2 (*Kcnq2*) α‐subunit present only in the type I hair cell (Table 2), whose contribution, however, was likely to be negligible given that linopirdine was ineffective on all type I hair cells tested (Figure 5). Therefore, the distinct current responses of type I and type II hair cells (specifically, the low‐voltage activation threshold of *I*_KL_) might stem from different combinations of the same K^+^ channel subunits in the two hair cell types. Alternatively, we might have failed to detect transcripts present at low expression levels. Furthermore, the importance of the K_V_7.2 α‐subunit might increase postnatally, because it has been identified in all vestibular hair cells of the chick utricle at P7 (Scheibinger et al., 2022).

Additional transcripts found only in the type I hair cell include that for *Chrna10* (Neuronal acetylcholine receptor subunit alpha‐10), that for *Fat4*, an atypical cadherin, and that for *Lfng* (Lunatic Fringe) (Table 2). Previously, *Chrna10* was found to be expressed in P7 chicken striolar type I and II hair cells (Scheibinger et al., 2022) and in mouse vestibular type II hair cells (Poppi et al., 2023). As far as *Fat4* is concerned, *Fat4* ^−/−^ mice display altered cochlear and vestibular morphology (Saburi et al., 2012). Finally, *Lfng*‐coded protein modulates Notch receptor affinity to Delta like ligand 1 and Jagged1 and is broadly expressed in the developing mouse cochlea, becoming progressively restricted to supporting cells (Zhang et al., 2000).

In contrast, the transcript for *Snap25* (Synaptosome associated protein 25) was found only in the type II hair cell (Table 2). Snap‐25 is required for normal exocytosis at auditory hair cell ribbon synapses (Calvet et al., 2022).

### 4.5 Discrepancies with the literature

*Agr3*, found here in both hair cell types, has been considered a marker of supporting cells in the utricle (Scheibinger et al., 2022; Zhu et al., 2019).

*Calb1* (Calbindin‐1), an intracellular Ca^2+^ binding protein, has been reported in chick type I and II hair cells located in the central region of the crista (Dechesne et al., 1988). The transcript for *Calb2* (Calbindin‐2; Calretinin), also an intracellular Ca^2+^‐binding protein, has been found in type I and II vestibular hair cells of the utricle of P7 chicks (Scheibinger et al., 2022). In our study, *Calb1* and *Calb2* transcripts were not detected in Patch‐seq experiments but were present in the whole‐organ transcriptome. This discrepancy might reflect differences in transcript detection sensitivity between the two approaches or variability in gene expression levels among individual hair cells.

*Gjb6* (gap junction protein) has been found in both hair cell types. However, in P7 chicks and mice it was found only in supporting cells (Burns et al., 2015; Scheibinger et al., 2022).

Otoferlin (*Otof*) is a crucial protein for Ca^2+^‐dependent synaptic vesicle exocytosis in vestibular type I and II hair cells (Dulon et al., 2009). *Otof* was previously reported to be expressed in utricle hair cells of P7 chicks (Scheibinger et al., 2022). In mice, *Otof* is expressed by type I hair cells (McInturff et al., 2018) although, according to Xu et al. (2025), it is expressed by all mouse hair cells at variable levels. In the present Patch‐seq study, *Otof* expression was found in the type I but not the type II hair cell. It is likely that *Otof* expression in the sequenced type II hair cell was below the detection threshold.

*Spp1* (Secreted phosphoprotein 1; previously known as osteopontin), has been found in the Patch‐sequenced type I and type II hair cells. In mice, it is expressed exclusively in mouse type I hair cells (McInturff et al., 2018).

## 5 Conclusion

Owing to its easy accessibility, the chicken embryo represents a valuable model for studying the development and pharmacology of ion channels in high vertebrates. In the present study, we report, for the first time, that type I and II hair cells of the chicken embryo, like their mouse counterparts (Manca et al., 2021; Martin et al., 2024), express the α‐subunits Ca_V_1.3 and K_V_1.8. Furthermore, we provide the molecular identity of other ion channels that also appear to be expressed in mouse hair cells, at least according to single‐cell RNA‐sequencing analysis of cells dissociated from whole vestibular sensory epithelia and identified by cell‐specific markers and cluster analysis (McInturff et al., 2018; Wilkerson et al., 2021; Xu et al., 2025).

Defining the nature of ion channels expressed by vestibular hair cells is essential for assessing the cause of vestibular disorders of peripheral origin and developing effective pharmacological treatments. For example, patients with the p.W276S mutation of *KCNQ4* (K_V_7.4) show abnormal clinical vestibular function tests (De Leenheer et al., 2002). In mice, null mutation of *Kcna10* (K_V_1.8) causes significant vestibular dysfunction (Lee et al., 2013); the *Kcna10*(TM45) targeted allele might be a model of human non‐syndromic vestibulopathy (Lee et al., 2013).

We recently demonstrated that pimozide, a conventional antipsychotic drug of the diphenylbutylpiperidine class with vestibular side effects (Roessner et al., 2022), substantially increases the amplitude of a sustained outwardly rectifying K^+^ current (*I*_KV_) in type II hair cells, but not in type I hair cells, of the chicken embryo and even of the adult mouse (Giunta et al., 2023, 2024). The molecular target of pimozide, however, has not been identified. Because the outward K^+^ current in adult mouse type I hair cells consists primarily of *I*_KL_, which is carried by K_V_1.8 subunits assembled in a homomeric channel (Martin et al., 2024), this subunit should not be affected by pimozide. The non‐*I*_KL_ current in type II hair cells should include the K_V_1.6 and the K_V_4.2 subunits. Because the K_V_4.2 subunit mediates a rapidly inactivating A‐type outward K^+^ current, the agonistic effect of pimozide on *I*_KV_ in type II hair cells should involve the K_V_1.6 subunit. As mentioned above, the K_V_1.6 subunit has been reported in mouse crista hair cells (Wilkerson et al., 2021) and might therefore represent a target of pimozide in humans as well.

## AUTHOR CONTRIBUTIONS

Patch‐clamp whole‐cell experiments were performed at the Department of Brain and Behavioural Sciences, University of Pavia, Pavia, Italy. RT‐PCR experiments were performed at the Department of Molecular Medicine, University of Pavia, Pavia, Italy. Whole‐organ and single‐cell sequencing was performed at the Università Vita‐Salute San Raffaele, Milan, Italy. Cheli Giulia, Giunta Roberta, Stella Francesca, Guidi Eleonora, Laforenza Umberto, Polimeni Mariarosa, Russo Giancarlo, Borgo Francesca and Lazarevic Dejan: data acquisition, analysis and interpretation; revision of the manuscript. Masetto Sergio: conception and design of the study; funding acquisition; analysis and interpretation of data; drafting the work. All authors approved the final version of the manuscript and agree to be accountable for all aspects of the work in ensuring that questions related to the accuracy or integrity of any part of the work are appropriately investigated and resolved. All persons designated as authors qualify for authorship, and all those who qualify for authorship are listed.

## CONFLICT OF INTEREST

None declared.

## GENERATIVE AI STATEMENT

No generative AI tools were used in the preparation of this manuscript.

## Data Availability

Electrophysiological data will be made available upon reasonable request. Whole‐organ and single‐cell transcriptomics data are available at NCBI Gene Expression Omnibus (GEO) data repository (GSE336620).

## 1

Statistics and other numerical data as specified in the text are provided in the Appendix (Tables A1, A2, A3, A4, A5).

**TABLE A1:** Numerical values underlying Figure 2a,b.

Voltage (mV)	CTRL (pA; mean ± SD)	LPD 10 µM (pA; mean ± SD)	WASH (pA; mean ± SD)	CTRL vs. LPD (adjusted *P*‐value)	CTRL vs. WASH (adjusted *P*‐value)	LPD vs. WASH (adjusted *P*‐value)
−70	0	0	0	–	–	–
−60	0.0554 ± 0.0341	0.052 ± 0.0373	0.0583 ± 0.0234	0.8920	0.9699	0.8969
−50	0.1561 ± 0.0663	0.1636 ± 0.0752	0.1841 ± 0.0868	0.9198	0.6612	0.8360
−40	0.3503 ± 0.0983	0.365 ± 0.0889	0.4066 ± 0.148	0.9268	0.6321	0.6931
−30	0.6454 ± 0.0486	0.6183 ± 0.1325	0.6819 ± 0.221	0.8295	0.8833	0.5968
−20	1 pA ± 0	0.9176 pA ± 0.1746	1.0034 ± 0.282	0.3041	0.9994	0.5270

*Note*: Values are the mean ± SD of type II hair cell (*n* = 12) normalized peak current recorded at each test potential in control conditions (CTRL), after linopirdine (LPD) perfusion and after washout (WASH). Statistical analysis was performed using a mixed‐effects model followed by Tukey's multiple‐comparisons test. Reported *P*‐values are Tukey‐adjusted exact *P*‐values.

**TABLE A2:** Numerical values underlying Figure 2c,d.

Voltage (mV)	CTRL (pA; mean ± SD)	LPD 10 µM (pA; mean ± SD)	WASH (pA; mean ± SD)	CTRL vs. LPD (adjusted *P*‐value)	CTRL vs. WASH (adjusted *P*‐value)	LPD vs. WASH (adjusted *P*‐value)
−70	0	0	0	–	–	–
−60	0.0106 ± 0.0069	0.0127 ± 0.0079	0.012 ± 0.0081	0.6988	0.7089	0.9740
−50	0.0273 ± 0.016	0.0351 ± 0.0191	0.0327 ± 0.019	0.2751	0.3988	0.9063
−40	0.0601 ± 0.0328	0.0712 ± 0.03	0.0744 ± 0.0278	0.3939	0.2590	0.9196
−30	0.1173 ± 0.0495	0.1328 ± 0.0372	0.147 ± 0.042	0.4851	0.2179	0.6024
−20	0.2195 ± 0.0576	0.2277 ± 0.045	0.2414 ± 0.061	0.8606	0.6441	0.7995
−10	0.3531 ± 0.056	0.3351 ± 0.0521	0.3464 ± 0.0749	0.5221	0.9636	0.8870
0	0.4924 ± 0.0512	0.4451 ± 0.0543	0.4528 ± 0.0843	0.0421	0.3331	0.9467
+10	0.6285 ± 0.0417	0.5532 ± 0.0587	0.5621 ± 0.0962	0.0052	0.1229	0.9504
+20	0.7567 ± 0.033	0.6628 ± 0.0659	0.6707 ± 0.1023	0.0031	0.0585	0.9688
+30	0.8807 ± 0.0192	0.7643 ± 0.0753	0.7792 ± 0.1043	0.0013	0.0471	0.9234
+40	1 ± 0	0.8662 ± 0.0895	0.8875 ± 0.11	0.0008	0.0511	0.8892

*Note*: Values are the mean ± SD of type II hair cell (*n* = 12) normalized steady‐state current recorded at each test potential in control conditions (CTRL), after linopirdine (LPD) perfusion and after washout (WASH). Statistical analysis was performed using a mixed‐effects model followed by Tukey's multiple‐comparisons test. Reported *P*‐values are Tukey‐adjusted exact *P*‐values.

**TABLE A3:** Numerical values underlying Figure 5a,b.

Voltage (mV)	CTRL (pA; mean ± SD)	LPD 10 µM (pA; mean ± SD)	WASH (pA; mean ± SD)	CTRL vs. LPD (adjusted *P*‐value)	CTRL vs. WASH (adjusted *P*‐value)	LPD vs. WASH (adjusted *P*‐value)
−70	0	0	0	–	–	–
−60	0.0602 ± 0.0165	0.0671 ± 0.0209	0.0669 ± 0.0216	0.5493	0.5257	0.9953
−50	0.1367 ± 0.0244	0.1456 ± 0.0355	0.1423 ± 0.0412	0.7145	0.8734	0.3918
−40	0.2214 ± 0.0308	0.2298 ± 0.0484	0.2184 ± 0.0541	0.8678	0.9811	0.0359
−30	0.309 ± 0.0389	0.3177 ± 0.0575	0.2982 ± 0.0611	0.9072	0.8384	0.0125
−20	0.4029 ± 0.0418	0.4076 ± 0.0638	0.3809 ± 0.0631	0.9793	0.5885	0.0031
−10	0.4992 ± 0.0425	0.4988 ± 0.0707	0.4668 ± 0.0635	0.9999	0.4327	0.0063
0	0.599 ± 0.0402	0.5897 ± 0.0784	0.5488 ± 0.0609	0.9513	0.1935	0.0081
+10	0.699 ± 0.0356	0.6801 ± 0.0817	0.6319 ± 0.0589	0.8304	0.1036	0.0102
+20	0.8053 ± 0.0284	0.77 ± 0.0857	0.7131 ± 0.0552	0.5264	0.0320	0.0153
+30	0.9052 ± 0.0145	0.8575 ± 0.091	0.7885 ± 0.0493	0.3459	0.0062	0.0207
+40	1 ± 0	0.9396 ± 0.0921	0.8626 ± 0.0498	0.2214	0.0019	0.0497

*Note*: Values are the mean ± SD of type I hair cell (*n* = 8) normalized peak current recorded at each test potential in control conditions (CTRL), after linopirdine (LPD) perfusion and after washout (WASH). Statistical analysis was performed using a mixed‐effects model followed by Tukey's multiple‐comparisons test. Reported *P*‐values are Tukey‐adjusted exact *P*‐values.

**TABLE A4:** Numerical values underlying Figure 5c,d.

Voltage (mV)	CTRL (pA; mean ± SD)	LPD 10 µM (pA; mean ± SD)	WASH (pA; mean ± SD)	CTRL vs. LPD (adjusted *P*‐value)	CTRL vs. WASH (adjusted *P*‐value)	LPD vs. WASH (adjusted *P*‐value)
−70	0	0	0	–	–	–
−60	0.0728 ± 0.0345	0.0835 ± 0.0379	0.0876 ± 0.0384	0.4466	0.3684	0.5480
−50	0.164 ± 0.0523	0.1778 ± 0.0653	0.1823 ± 0.0748	0.6682	0.6267	0.6318
−40	0.2621 ± 0.0582	0.2733 ± 0.0835	0.2716 ± 0.0978	0.8818	0.9371	0.9509
−30	0.355 ± 0.0634	0.3624 ± 0.0986	0.3537 ± 0.1088	0.9667	0.9991	0.4073
−20	0.4478 ± 0.0637	0.4487 ± 0.109	0.431 ± 0.112	0.9996	0.8812	0.0967
−10	0.5439 ± 0.0557	0.5337 ± 0.1139	0.5061 ± 0.1109	0.9546	0.5579	0.0529
0	0.6430 ± 0.0459	0.6158 ± 0.1189	0.5808 ± 0.1107	0.7645	0.2654	0.0426
+10	0.7385 ± 0.0358	0.6945 ± 0.1187	0.6533 ± 0.1084	0.5050	0.1093	0.0322
+20	0.8328 ± 0.0257	0.7709 ± 0.1178	0.7239 ± 0.1012	0.2938	0.0422	0.0327
+30	0.9193 ± 0.0121	0.8435 ± 0.1166	0.7868 ± 0.0898	0.1976	0.0171	0.0439
+40	1 ± 0	0.909 ± 0.1108	0.8569 ± 0.0779	0.1176	0.0084	0.0704

*Note*: Values are the mean ± SD of type I hair cell (*n* = 8) normalized steady‐state current recorded at each test potential in control conditions (CTRL), after linopirdine (LPD) perfusion and after washout (WASH). Statistical analysis was performed using a mixed‐effects model followed by Tukey's multiple‐comparisons test. Reported *P*‐values are Tukey‐adjusted exact *P*‐values.

**TABLE A5:** Numerical values underlying Figure 6.

Gene	Fold change (mean ± SD)	*P*‐value
*CABI*	1.98 ± 1.83	0.4539
*CARE*	0.98 ± 0.74	0.4222
*MYOS*	1.77 ± 1.17	0.4419
*KCNQ2*	0.40 ± 0.17	0.0013
*KCNQ4*	n.d.	–
*kcnq5*	0.31 ± 0.29	0.0068
*kcna3*	0.80 ± 0.66	0.1569
*kcna4*	1.06 ± 0.94	0.9696
*kcna10*	11.83 ± 13.86	0.0040
*kcnb1*	0.83 ± 0.65	0.1851
*kcng4*	3.12 ± 2.53	0.0720
*kcnh7*	0.88 ± 0.84	0.3226
*kcnj2*	11.54 ± 9.61	0.0002
*kcnj5*	0.54 ± 0.47	0.0226
*kcnj11*	1.64 ± 1.80	0.5747
*hcn1*	0.83 ± 0.71	0.2640

*Note*: Relative gene expression is presented as the fold change (2^−ΔΔ^ *^Ct^*) relative to embryonic day 16 (baseline = 1) and expressed as the mean ± SD (*n* = 4 independent experiments). Statistical analyses were performed on Δ*Ct* values using Student's two‐tailed unpaired *t*‐tests. Exact *P*‐values are reported.
